# Systemic use of tumor necrosis factor alpha as an anticancer agent

**DOI:** 10.18632/oncotarget.344

**Published:** 2011-10-27

**Authors:** Nicholas J. Roberts, Shibin Zhou, Luis A. Diaz, Matthias Holdhoff

**Affiliations:** ^1^Ludwig Center for Cancer Genetics and Therapeutics, The Sidney Kimmel Comprehensive Cancer Center, The Johns Hopkins University, Baltimore, MD 21231, USA

**Keywords:** cancer, chemotherapy, TNF, drug combinations, oncotarget

## Abstract

Tumor necrosis factor-α (TNF-α) has been discussed as a potential anticancer agent for many years, however initial enthusiasm about its clinical use as a systemic agent was curbed due to significant toxicities and lack of efficacy. Combination of TNF-α with chemotherapy in the setting of hyperthermic isolated limb perfusion (ILP), has provided new insights into a potential therapeutic role of this agent. The therapeutic benefit from TNF-α in ILP is thought to be not only due to its direct anti-proliferative effect, but also due to its ability to increase penetration of the chemotherapeutic agents into the tumor tissue. New concepts for the use of TNF-α as a facilitator rather than as a direct actor are currently being explored with the goal to exploit the ability of this agent to increase drug delivery and to simultaneously reduce systemic toxicity.

This review article provides a comprehensive overview on the published previous experience with systemic TNF-α. Data from 18 phase I and 10 phase II single agent as well as 18 combination therapy studies illustrate previously used treatment and dose schedules, response data as well as the most prominently observed adverse effects. Also discussed, based on recent preclinical data, is a potential future role of systemic TNF-α in combination with liposomal chemotherapy to facilitate increased drug uptake into tumors.

## Introduction

TNF-α was discovered in 1975 and subsequently cloned in 1984 [1, 2] and has been the focus of considerable interest as an anticancer agent. Initial enthusiasm for TNF-α as a systemic therapeutic agent stemmed from the observation that it could induce hemorrhagic necrosis in the tumors of bacillus Calmette-Guérin (BCG)-primed and endotoxin treated mice [1]. Recombinant human TNF-α (rhTNF-α) has been tested as a systemic treatment in several phase I and phase II clinical trials. These trials, conducted in the 1980s and 1990s, used TNF-α as both a single agent and in combination with other cytokines or chemotherapeutics. However, the initial enthusiasm for the development of TNF-α as a systemic treatment has waned in the face of significant toxicities and a lack of evidence for therapeutic benefit. Nevertheless, these studies have provided valuable data regarding the toxicity profile and pharmacological properties of systemically delivered TNF-α. More encouraging is the current clinical use of combination TNF-α with chemotherapeutic agents in the setting of hyperthermic isolated limb perfusion in limb-threatening soft tissue sarcomas and in-transit melanoma [3]. Here we review the phase I and phase II clinical trials of systemic use of TNF-α, the toxicities and responses observed, and highlight recent scientific advances that hint at reduced systemic toxicities and augmentation of the antitumor responses seen with this agent. Specifically, the recently identified vascular effects of TNF-α that lead to a targeted intra-tumoral enrichment of liposomes and macromolecules through an enhanced-enhanced permeability and retention effect (E^2^PR) [4-6].

## BIOLOGY OF TNF-α

TNF-α is a 23 kilodalton (kDa) type II transmembrane protein arranged in stable homotrimers. A 51 kDa soluble homotrimeric cytokine is derived from the transmembrane form via proteolytic cleavage by the metalloprotease TNF-α converting enzyme (TACE) [7]. TNF-α is primarily produced by macrophages, but also by a variety of other cells, including NK cells, T lymphocytes, smooth muscle cells, fibroblasts and others [8]. Release of TNF-α occurs in response to inflammatory stimuli and cytokines including peptidoglycan, lipopolysaccharide and other bacterial components [9]. Two receptors exist for TNF-α: 1). Tumor necrosis factor receptor 1 (TNFR1), which preferentially binds soluble TNF-α and is found almost ubiquitously on the surface of cells, and 2). Tumor necrosis factor receptor 2 (TNFR2), which is found on cells of the hematopoietic lineage and which has specificity for the transmembrane form of TNF-α [10]. The resulting biological effect of TNF-α binding to its receptor is depending on the type of receptor activated and the cellular state during activation. Stimulation of TNFR1 activates downstream inflammatory mediators through AP1, MAPK and NF-kB pathways [11]. The balance of activation of these pathways by TNF-α is critical in determining whether a cell undergoes apoptosis as a late stage event in TNF-α stimulation. For instance, in acute myeloid leukemia (AML), NF-kB dependent induction of HO-1 underlies resistance to TNF-α induced apoptosis [12]. Similarly, inhibition of NF-kB with concurrent TNF-α stimulation results in caspase activation and apoptosis [13]. Conversely, the biological role and downstream effects of TNFR2 stimulation are more poorly understood. TNFR2 can be up-regulated by cytokine stimulation and also mediate a variety of downstream inflammatory mediators [14].

## PRECLINICAL EVIDENCE FOR TNF-α AS AN ANTICANCER AGENT

After the initial observation that TNF-α induced hemorrhagic necrosis of tumors in mice treated with BCG and endotoxin [1], the potential of TNF-α as a therapeutic agent was intensely studied in *in vitro* and *in vivo* studies. These studies highlighted the possible role of TNF-α as an anticancer agent and galvanized support for the numerous phase I and phase II studies that followed.

*In vitro* studies demonstrated that TNF-α had a growth inhibitory effect on SV40-tranformed human mammary epithelia cells and a cytotoxic effect on breast cancer cell lines. Interestingly, there was no effect on normal human mammary epithelial cells [15]. Similarly, TNF-α showed a cytostatic effect on hepatoma cells while having little effect on non-tumorigenic liver cells [16]. Intriguingly, Sugarman and colleagues showed that the cytostatic and cytotoxic effects of TNF-α were cell line specific, with only a proportion of tumor cell lines responding to TNF-α [17]. Comparison of the cytostatic and cytotoxic effect of TNF-α against a wide range of tumor types demonstrated that approximately a quarter of tumors (28%) are sensitive to the effects of TNF-α and that this sensitivity was greater in colorectal and lung cancers [18]. *In vivo*, TNF-α has shown activity against a wide variety of murine tumor types and human tumor xenografts [19-21]. Taken together, the *in vitro* and *in vivo* data were suggestive that TNF-α had the potential to be highly specific anti-cancer therapy, with activity against a number of tumor types.

Preclinical evidence also suggested a synergistic effect of TNF-α with a variety of chemotherapeutics *in vitro* and *in vivo*. TNF-α has been shown to enhance the cytotoxicity of DNA topoisomerase inhibitors actinomycin D, adriamycin, and etoposide against murine bladder tumor cell line (MBT-2) in *in vitro* and *in vivo* models [22]. The enhancing effect of TNF-α was not observed with other cytotoxic agents, such as: bleomycin, hydroxyurea, cisplatin, mitomycin C, vincristine and vinblastine. The timing of TNF-α treatment in relation to chemotherapy seems important with studies suggesting that the optimal time for TNF-α therapy is 48 hours prior to initiation of chemotherapy [23]. Interestingly, there are likely two mechanisms that underlie the importance of timing in regards to TNF-α treatment. Firstly, inhibitors of transcription, such as actinomycin D and flavopiridol, are used before or at the time of TNF-α treatment and block NF-kB pathway activation, sensitizing cells to the effects of TNF-α [24]. Secondly, inhibitors of topoisomerase II can be given at the time of, or after TNF-α and increase the sensitivity of TNF-α resistant cancer cell lines to TNF-α [25].

TNF-α also demonstrated enhanced antitumor effects in vitro when used in combination with other cytokines [26]. Induction of TNF-α receptors rather than an increased affinity of already present receptors explained the effect of IFN-γ on TNF-α binding [26, 27]. Similarly, *in vitro* and* in vivo* studies using the TNF-α resistant melanoma cell line B16BL6 demonstrated that IFN-γ sensitizes cancer cells to the effects of TNF-α, inducing necrosis and tumor response, which were previously absent [28]. Later, investigators showed that TNF-α induced synergistic growth inhibition against pancreatic cancer cell lines when combined with interferon alpha (IFN-α) and IFN-γ [29]. TNF-α and IFN-γ act against many other cancer cell lines as well. Orita *et al*. [30] tested TNF-α and IFN-γ on 23 cell lines in vitro and demonstrated that the combination acts synergistically, showing cytostatic and cytotoxic effects on cell lines previously resistant to TNF-α and IFN-γ individually.

Combinations of TNF-α with IFN-α and IL-2 also showed synergistic cytotoxic and cytostatic effects *in vitro* and *in vivo*. Concomitant TNF-α and IFN-α in a murine lung metastasis model significantly increased survival [31]. TNF-α and IL-2 in murine models with leukemia, mastocytoma, melanoma, lymphoma and sarcoma cell lines also demonstrate combinatorial effects and systemic immunological memory [32, 33].

The combination of TNF-α and radiotherapy has been less extensively studied. Investigation of the interaction of TNF-α and radiation in 14 human tumor cells lines demonstrated synergistic or additive cytotoxicity with the maximum effect when TNF-α was given 4-12 hours before irradiation [34]. The mechanism of this synergism is thought to be due to the induction of oxygen free radical species and resulting DNA damage.

## CLINICAL TRIALS OF SYSTEMIC RECOMBINANT HUMAN TNF-α

### Systemic rhTNF-α as a single agent

Numerous phase I and phase II studies have been conducted to ascertain the toxicity profile and efficacy of systemic TNF-α. Studies have encompassed a wide range of tumor types in both adult and pediatric patients. In the majority of phase I and phase II studies, TNF-α was administered as an intravenous bolus injection or infusion. However, a few phase I studies have evaluated TNF-α with subcutaneous or intramuscular administration.

Phase I studies conducted with TNF-α are detailed in Table 1 [35-52]. Eighteen phase I studies were conducted and published with rhTNF-α as a single agent systemic therapy, enrolling between 19 and 62 patients per study. Study design varied with single dose of rhTNF-α, multiple dosing (daily to every three weeks) and continuous infusion (one to five day duration) being tested. Overall, it appears that a systemic TNF-α dose of 150-200 μg/m^2^, given as a 30 minute intravenous infusion was identified as MTD in several studies. Dose-limiting toxicities (DLT) as well as other side effects that were observed seemed to have been universal and in most cases reasonably well tolerated and reversible. Common DLTs included: hypotension, thrombocytopenia, leukopenia, neurotoxicity, fever, nausea/vomiting, as well as general symptoms of malaise and weakness (Table 2). Other pathological sequelae of a transient hypovolemic episode, including transient elevation of liver enzymes, were reported. Tumor responses however, when used as a single agent, even with more intense treatment schedules, were rare.

**Table 1:** Phase I studies with single agent rhTNF-α

Study	Total number of patients	Tumor Type	Dose TNF-α^a^	Schedule	ORR^b^	MTD	Dose Limiting Toxcities
**Chapman 1987 [35]**	13	Advanced cancer	1 - 200 μg/m^2^ for (IV bolus) and 5 - 250 μg/m^2^ (SQ)	Twice weekly alternating SQ/IV rhTNF-α every week for 4 weeks	8%	NR	Hypotension. Local tissue reaction. Nausea. Vomiting. Neurotoxicity.
**Creaven 1987 [36]**	29	Advanced cancer - solid tumors	1 × 10^4^ - 48 × 10^4^ units/m^2^	Three doses 3 weeks apart	0%	48 × 10^4^ units/m^2^	Hypotension.
**Kimura 1987 [37]**	33	Advanced cancer - solid tumors	1 × 10^5^ - 16 x10^5^ units/m^2^	One dose	0%	5x 10^5^ units/m^2^	Hypotension. Thrombocytopenia. Hepatotoxicity.
**Creagan 1988 [38]**	27	Advanced cancer - solid tumors	5 - 200 μg/m^2^/day	Daily for 5 consecutive days every 2-3 weeks	4%	150 μg/m^2^	Hypotension. Rigors. Phlebitis.
**Feinberg 1988 [39]**	39	Metastatic cancer	5 - 250 μg/m^2^/day	Daily for five consecutive days every two weeks for 8 weeks. 30 minute vs. 4 hour infusion	0%	200 μg/m^2^/day	Hypotension. Nausea. Vomiting. Myalgias. Fatigue.
**Sherman 1988 [40]**	19	Advanced cancer - solid tumors	0.5 × 10^4^ - 3.0 × 10^5^ units/m^2^/day	5-day continuous infusion every 4 weeks	0%	3.0 × 10^5^ units/m^2^/d.	Thrombocytopenia. Leukopenia.
**Spriggs 1988 [41]**	50	Advanced cancer	4.5 - 645 μg/m^2^	Continuous infusion over 24 hours every 3 weeks	2%	636 μg/m^2^	Hypotension.
**Taguchi 1988 [42]**	53^c^	Malignant tumors	0.1 × 10^6^ - 5 × 10^6^units/dose (IV); 0.1 × 10^6^ - 2 × 10^6^units/dose (IT)	One dose for week 1, then three times a week for week 2-7	5%	1 × 10^6^ units/dose^d^	Hypotension.
**Creaven 1989 [43]**	33	Advanced cancer	5 - 80 × 10^4^ units/m^2^/day	Daily for 5 consecutive days	6%	60 × 10^4^ units/m^2^/day	Hypotension. Hepatotoxicity.
**Jakubowski 1989 [44]**	19	Advanced cancer	5 - 200 μg/m^2^/day (IM)	Daily for 5 consecutive days every 2 weeks	0%	150 μg/m^2^/day	Local injection site reaction. Leukopenia. Thrombocytopenia. Hepatoxicity. Neurotoxicity.
**Wiedenmann 1989 [45]**	15	Advanced cancer - adenocarcinoma	40 - 400 μg/m^2^	Continuous infusion over 24 hours once or twice weekly for 8 weeks	0%	200 μg/m^2^	Thrombocytopenia. Fever. Cholls. Fatigue. Myalgia.
**Gamm 1991 [46]**	62	Advanced cancer	2.5 - 200 μg/m^2^	Twice daily for 5 consecutive days every two weeks for 8 weeks	6%	150 μg/m^2^/dose	Hypotension. Hepatotoxicity.
**Krigel 1991 [47]**	27	Advanced cancer - solid tumors	8.5 - 1000 μg/m^2^	100% dose on day 1, then 20% of initial dose on day 8 - day 12 repeated every 2 weeks	0%	267 μg/m^2^ (initial dose) and 160 μg/m^2^ (subsequent daily dosing)	Hypotension. Hemorrhagic gastritis.
**Logan 1991 [48]**	24	Advanced cancer - solid tumors	40 - 240 μg/m^2^	100% dose on day 1, then daily dosing on day 8 - day 12 repeated every 3 weeks	NR	NR	NR
**Schiller 1991 [49]**	53	Advanced cancer	5 - 275 μg/m^2^	Three times a week for 4 weeks	2%	225 μg/m^2^	Hypotension. Fatigue. Nausea.
**Mittelman 1992 [50]**	19	Advanced cancer - solid tumors	40 - 200 μg/m^2^	24-hour infusion on day 1 followed by 120-hour infusion day 8 - day 12 repeated every 3 weeks	0%	160 μg/m^2^	Hematologic toxicity. Neurotoxicity.
**Furman 1993 [51]**	27	Pediatric advanced cancer	100 - 350 μg/m^2^/day	Daily for 5 consecutive days every two weeks	4%	300 μg/m^2^/day	Cardiotoxicity. Hypotension. Hepatotoxicity.
**Braczkowski 1998 [52]**	21	Advanced cancer - solid tumors	75 - 150 μg/day	Daily for 5 consecutive days every two weeks	48%	N/A	NR
^a^All Studies used intravenous infusion for delivery of TNF-α. unless otherwise indicated. ^b^Ojective response rate calculated using number of patients evaluable for response where available. ^c^Intravenous (43 patients) and intratumoral (10 patients). ^d^IV dose only. TNF-α - tumor necrosis factor alpha. ORR - objective response rate. MTD - maxium tolerated dose. IV - intravenous. IM - intramuscular. SQ - subcutaneous. IT - intratumoral.							

**Table 2:** Side effects of single agent rhTNF-α

	Side Effect
**Very Common**	Hypotension
	Hepatotoxicity
**Common**	Nausea
	Neurotoxicity
	Vomiting
	Chills
	Fatigue
	Fever
	Leukopenia
	Rigors
	Thrombocytopenia
	Cardiotoxicity
	Gastrointestinal toxicity
	Myalgia
	Anemia
	Dyspnea
	Hematologic toxicity
	Local tissue reaction
	Pain
	Pulmonary toxicity
**Uncommon**	Anorexia
	Arthropathy
	Coagulopathy
	Constituitive symptoms
	Diarrhea
	Fever
	Hematuria
	Hemorrhagic gastritis
	Hyperglycemia
	Hypertension
	Intracranial hemorrhage
	Lethargy
	Leukocytosis
	Lymphopenia
	Neuropathy
	Phlebitis
	Renal toxicity
	Tachycardia
	Vascular thrombosis
Side effects to systemic rhTNF-α monotherapy observed as a dose-limiting toxicity or ≥ grade 3 toxicity in a phase I or phase II study. Very common side effect seen in > 10 studies. Common side effect seen in between 2 and 10 studies. Uncommon side effect seen in 1 study.	

Phase II studies using systemically administered rhTNF-α are detailed in Table 3 [53-62]. Studies typically investigated advanced/metastatic cases of: colorectal cancer, breast cancer, pancreatic cancer, malignant melanoma and renal cell carcinoma. The majority of studies involved a small number of cases (16-26), an exception being a phase II study of various malignancies that enrolled 147 patients [59]. Study design varied, with 150-200 μg/m^2^ given as a 30 minute intravenous infusion daily for 3-5 days and repeated every 1-4 weeks being commonly employed. In all studies, tumor responses were rare and when they did occur, only partial responses were observed. In the largest study of 147 cancer patients treated with 150μg/m^2^ for 5 days every other week, only 1 partial remission was noted while 13% of patients experienced a grade 4 or greater toxicity. The most serious toxicities included respiratory failure and coagulopathies. Other, less serious and more common side effects reported include: hypotension (31%), leukopenia (38%), thrombocytopenia (13%), fever / chills (69%), headache (25%), nausea / vomiting (69%) and hepatopathy (10%). However, compared to other phase II studies this regimen was fairly dose-dense which may have increased the significant toxicity observed.

**Table 3:** Phase II studies with single agent rhTNF-α

Study	Total number of patients	Tumor Type	Dose TNF-α^a^	Schedule	Maximum Number of Cycles	ORR^b^	Major Reported Toxicities^c^
**Lenk 1989 [53]**	22	Advanced cancer - solid tumors	683 - 956 μg/m^2^	Weekly	6	NR	Hypotension. Leukocytosis. Hepatotoxicity. Nausea. Vomiting.
**Heim 1990 [54]**	15	Advanced colorectal cancer	3 × 10^5^ U/m^2^/day	Daily for days 1-3 every 2 weeks	4	9%	Dyspnea. Fever. Leucopenia.
**Kemeny 1990 [55]**	16	Advanced colorectal cancer	100-150 μg/m^2^/day	100 μg/m2/day BID on day one. 100 μg/m2/day BID on days 2-5. Repeat every other week	4	NR	Gastrointestinal toxicity. Neurotoxicity. Chills. Pain. Hypotension. Hypertension. Leukopenia. Hepatoxicity. Vascular thrombosis.
**Whitehead 1990 [56]**	25	Metastatic colorectal cancer	150 μg/m^2^/day	Daily for 5 days every 2 weeks	4	0%	Chills. Nausea. Vomiting. Anemia. Hepatoxicity.
**Brown 1991 [57]**	26	Pancreatic adenocarcinoma	150 μg/m^2^/day	Daily for 5 days every 2 weeks	7	NR	Fever. Rigor. Nausea/vomiting/anorexia. Hypotension. Hyperglycemia. Anemia. Dyspnea. Hepatoxicity. Coagulopathy. Tachycardia.
**Budd 1991 [58]**	22	Metastatic breast cancer	150 μg/m^2^/day	Daily for 5 days every 2 weeks	4	0%	Hypotension. Diarrhea. Leukopenia. Hepatotoxicity. Intracranial hemorrhage.
**Hersh 1991 [59]**	147	Metastatic malignancies	150 μg/m^2^/day	Daily for 5 days every 2 weeks	4	1%	Hematological toxicity. Gastrointestinal toxicity. Renal toxicity. Hepatotoxicity. Cardiovascular toxicity. Chills/fever. Lethargy. Neurotoxicity. Pulmonary toxicity.
**Feldman 1992 [60]**	21	Malignant melanoma	150 μg/m^2^/day	Daily for 5 days every 2 weeks for 4 cycles, then every three weeks	4+	5%	Fever. Chills. Nausea. Vomiting. Hypotension. Hepatotoxicity. Constituitive symptoms.
**Skillings 1992 [61]**	26	Metastatic renal cell carcinoma	150 μg/m^2^/day	Daily for 5 days every other week for 4 weeks	11	9%	Cardiovascular toxicity. Hematuria. Fatigue. Neurotoxicity. Rigors. Pain. Pulomary Toxicity. Gastrointestinal toxicity.
**Muc-Wierzgon 1996 [62]**	16	Advanced gastrointestinal cancers	150 μg/m^2^/day	Daily for 5 days every 2 weeks	6	NR	Fever. Rigor. Hypotension. Fatigue. Neuropathy. Myalgia. Arthropathy. Lymphopenia.
^a^ All Studies used intravenous infusion for delivery of TNF-α. unless otherwise indicated. ^b^ Ojective response rate calculated using number of patients evaluable for response where available.^c^ Grade 3 or greater toxicities. TNF-α - tumor necrosis factor alpha. ORR - objective response rate. NR - not reported in study.							

### Systemic rhTNF-α in combination with chemotherapeutics

Phase I and II studies that investigated the safety and efficacy of systemic rhTNF-α combined with carmustine [3], actinomycin D [63, 64], carboplatin and etoposide [65], dactinomycin [66] and doxorubicin [67] have been reported and are detailed in Table 4. In all trials, intravenous rhTNF-α was given concurrently or sequentially to chemotherapeutics on multiple days and treatments being repeated for a number of cycles. Dose of intravenous rhTNF-α ranged from 88-200μg/m^2^ and was similar to the dose used for rhTNF-α monotherapy. In one study of rhTNF-α and BCNU in advanced melanoma, a response rate of 20% was seen with BCNU alone compared to 10.5% with BCNU and rhTNF-α [3]. Additionally, treatment of recurrent or refractory Wilms tumor with dactinomycin and rhTNF-α resulted in a 15.8% response rate [66]. However, while patients in this study were previously treated with dactinomycin, response to therapy was not conclusively due to the action of rhTNF-α. Together, trials of rhTNF-α combined with chemotherapeutics have failed to prove that the addition of rhTNF-α to the treatment regimen improved outcome.

**Table 4:** Studies of systemic TNF-α with chemotherapy

Study	Total Number of Patients	Tumor Type	Study Design	Chemotherapy	Dose of Chemotherapy/TNF-α^a^	Regimen	Maximum Number of Cycles	ORR^b^	MTD	Major Reported Toxicities^c^
**Jones 1992 [3]**	41	Advanced melanoma	Phase II	BCNU	200 mg/m^2^ BCNU ± 88 μg/m^2^ rhTNF-α	Daily for 5 days every 48 days	2	BCNU + rhTNF-α -10.5% BCNU - 20%	N/A	Hepatotoxicity. Leukopenia. Hematological toxicity. Rigor.
**Seibel 1994 [63]**	33	Pediatric cancer	Phase I	Actinomycin D	Actinomycin 15 μg/kg on first day; rTNF-α 0-240 μg/kg/day	Daily for 5 days every 3 weeks	8	7%	200-220 μg/m^2^/day × 5	Hepatotoxicity. Leukopenia. Thrombocytopenia. Stomatitis. Hypotension. Pumlonary toxicity.
**Sella 1995 [64]**	21	Metastatic prostate cancer	Phase I	Actinomycin D	Actinomycin 1300-400 μg/m^2^; rTNF-α starting at 5-60 μg/m^2^	IV actinomycin D followed by rTNF daily for 5 days every 4 weeks	NR	0%	400 μg/m^2^ Actinomycin D and 40 μg/m^2^ rTNF-α	Fatigue. Neutropenia. Thrombocytopenia. Respiratory toxicity. Neurotoxicity. Nausea. Vomiting.
**Yamamoto 2002 [65]**	10	Recurrent malignant astrocytomas	Phase II	Carboplatin And Etoposide	Carboplatin 400 mg/m^2^ (day 1). Etoposide 100 mg/m^2^ (days 1-3). TNF-SAM2 80x10^4^ U/m^2^ (day 7)	Maximum 5 doses over 2 weeks every 8-12 weeks	4	33%	N/A	Leukopenia
**Meany 2008 [66]**	21	Recurrent or refractory Wilms tumor	Phase II	Dactinomycin	Dactinomycin 15 μg/kg/d and rTNF 200 μg/kg/d	Daily for 5 days every 3 weeks	10	16%	0.8μg/m^2^ NGR-hTNF and 75mg/m^2^	Thrombocytopenia. Hepatopathy. Neutropenia. Leucopenia. Anemia. Myalgia, Lymphopenia. Hypotension. Hematuria. Stomatitis. Nausea. Neurologic. Bronchospasm. Peripheral capillary leak.
**Gregorc 2009 [67]**	15	Solid tumors	Phase I	Doxorubicin	NGR-hTNF (0.2-0.4-0.8-1.6 μg/m2) and doxorubicin (60-75 mg/m2)	Every 3 weeks	15	7%	N/A (low dose NGR-hTNF therapy)	No dose limiting toxicities observed. Neutropenia, Anemai, Leukopenia, Thrombocytopenia, Leukopenia, Lymphopenia, Neutropenic fever, pain, comiting, cough, anorexia, hepatopathy, acute myocarfial infarction, pulmonary embolism.
^a^ All Studies used intravenous infusion for delivery of TNF-α. ^b^ Ojective response rate calculated using number of patients evaluable for response where available. ^c^ Dose limiting toxicities for phase I studies and grade 3 or greater toxicities for phase II studies. TNF-α - tumor necrosis factor alpha. ORR - objective response rate. MTD - maximum tolerated dose. NR - not reported in study. N/A - not applicable for study design.										

### Systemic rhTNF-α in combination with cytokines

Many studies have combined systemic administration of rhTNF-α with other cytokines such as: IFN-γ [68-72], IL-2 [73-76] and IFN-α [76], and these are summarized in Table 5. In general, phase I studies showed a reduction in the MTD of rhTNF-α when used in combination with other cytokines for patients with advanced solid tumors. This was largely due to the overlap in toxicities of these cytokines, that is: hypotension, fever, thrombocytopenia, acute renal failure, anemia, cardiac arrhythmias and pulmonary edema. Disappointingly, few objective responses were reported and none of the combinations were tested in larger randomized phase II studies; most likely because of the toxicity associated with combined therapy and a lack of efficacy seen in the initial studies.

**Table 5:** Studies of systemic TNF-α with other cytokines

Study	Total Number of Patients	Tumor Type	Study Design	Cytokine	Dose of Cytokine/TNF-α	Route	Bolus Infusion	Regimen	Cycles	ORR^a^	MTD	Major Reported Toxicities^b^
**Demetri 1989 [68]**	38	Advanced cancer	Phase I	IFN-γ	IFN-γ (200 μg/m^2^/24hr); rhTNF-α (2-205 μg/m^2^/24hr)	IV	Infusion	24hr infusion of IFN-γ ; 24hr rhTNF-α infusion 12 hours after the start of IFN-γ	NR	6%	205 μg/m^2^ of rhTNF-α	Hypotension.
**Kurzrock 1989 [69]**	25	Metastatic cancer	Phase I	IFN-γ	IFN-γ (5-75 μg/m^2^/24hr); rhTNF-α (5-75 μg/m^2^/24hr)	IM	Bolus	Daily for 5 days every 2 weeks	2	0%	rTNF-α 75 μg and IFN-γ 50 μg	Dyspnea. Fatigue. Hyperthermia. Hypertensive encephalopathy - seizure. Thrombocytopenia.
**Fiedler 1991 [70]**	16	Colorectal cancer	Phase I/II	IFN-γ	TNF (50 μg/m^2^ IV); IFN- γ (100 μg SC)	IV	Infusion	Daily for 5 days every week	4	0%	N/A	Acute renal failure. Thrombocytopenia.
**Smith 1991 [71]**	36	Solid tumors	Phase I	IFN-γ	IFN-γ (10-100 μg/m^2^; rhTNF-α (10-100 μg/m^2^/24hr)	IM	Bolus	IFN-γ followed 5 minutes later by rhTNF-α every other day	0	NR	100 μg/m^2^ of IFN-gamma plus 50 μg/m^2^ of TNF-α	Fever. Thrombocytopenia.
**Yang 1991 [73]**	16	Non-small cell lung cancer	Phase I	IL-2	IL-2 (6 × 10^6^ IU/m^2^ IV); TNF-α (25-100 μg/m^2^/day IM)	IM	Infusion	Daily for 5 days every 3 weeks	2	8%	6 × 10^6^ IU/m^2^ of IL-2 plus TNF-α 50 μg/m^2^/day	Thrombocytopenia.
**Schiller 1992 [72]**	24	Advanced cancer	Phase I	IFN-γ	IFN-γ (100 μg/day ); TNF-α (25-100 μg/m^2^/day)	IV	Infusion	Three times a week	4	NR	50 μg/m^2^ TNF-α and 100 μg/m^2^ IFN-γ	Hypotension.
**Krigel 1995 [74]**	15	Metastatic cancer - solid tumors	Phase I	IL-2	TNF-α (160 μg/m^2^); rIL-2 (6-18 × 10^6^ IU/m^2^/day)	IV	Infusion	TNF-alpha infusion for 5 days followed by rIL-2 for 5 or 7 days every 3-4 weeks	2	14%	160 μg/m^2^ TNF-α and 18 × 10^6^ IU/m2/day rIL-2	Hypotension. Weight loss. Fatigue.
**Schiller 1995 [75]**	8	Non-small cell lung cancer	Pilot Study	IL-2	IL-2 (6 × 10^6^ IU/m^2^/day IV); TNF-α (50μg/m^2^/day IM)	IM	Bolus	IL-2 and TNF-α daily for 4 days every week for 3 weeks	2	0%	N/A	Pulmonary toxicity. Cardiac toxicity. Renal toxicity. Neurotoxicity.
**Schiller 1995 [75]**	7	Non-small cell lung cancer	Phase I	IL-2	IL-2 (6 × 10^6^ IU/m^2^/day IV); TNF-α (50 -150μg/m^2^/day IM)	IM	Bolus	IL-2 and TNF-α daily for 5 days every 2 weeks	9	0%	< 6 × 10^6^ IU/m^2^/day of IL-2 and 50 μg/m^2^/day of TNF-α	Pulmonary toxicity. Cardiac toxicity. Renal toxicity. Neurotoxicity.
**Eskander 1997 [76]**	18	Metastatic cancer	Phase I	IL-2 & IFN-α	IFN-α (9 × 10^6^ IU/m^2^/day IM or SC); IL-2 (1-3 × 10^6^ IU/m^2^/day IV); TNF-α (40 - 120 μg/m^2^ IV)	IV	Infusion	IFN-α weekly on days 1, 3 and 5 for 3 weeks. IL-2 weekly on days 1-5 for 3 weeks. TNF-α on days 1-5 for week 1	NR	0%	40-80 μg/m^2^/day as 2-hour infusion depending on regimen	Pulmonary toxicity. Cardiac toxicity. Gastrointestinal toxicity. Cytopenia.
^a^ Ojective response rate calculated using number of patients evaluable for response where available. ^c^ Dose limiting toxicities for phase I studies and grade 3 or greater toxicities for phase II studies. ORR - objective response rate. MTD - maximum tolerated dose. NR - not reported in study. N/A - not applicable for study design. IV - intravenous. IM - intramuscular. IFN-γ - interferon gamma. IL-2 - interleukin 2. IFN-α - interferon alpha. TNF-α - tumor necrosis factor alpha.												

### Systemic rhTNF-α in combination with radiotherapy

Three studies that combined rhTNF-α with external beam radiation have also been reported [77-79] and they are detailed in Table 6. The most recent studies combined radiotherapy with both rhTNF-α and the chemotherapeutic ranimustine for the treatment of malignant astrocytoma [79]. In these studies DLTs were not observed and consequently, the maximum tolerated dose of this regimen was difficult to ascertain. In addition, no synergy in terms of objective response was noted.

**Table 6:** Studies of systemic TNF-α with radiation +/- chemotherapy

Study	Total number of patients	Tumor Type	Chemotherapy	Study Design	Dose TNF-α^a^	Regimen	Cycles	ORR^b^	MTD	Major Reported Toxicities^c^
**Hallahan 1995 [77]**	31	Advanced cancer	N/A	Phase I	TNF-alpha 10-150 μg/m^2^; radiation (150-300cGy/day; 30-60Gy total)	TNF-alpha given 4 hours prior to radiotherapy	N/A	40%	150 μg/m^2^	NR
**Fukushima 1998 [78]**	23	Malignant astrocytomas and glioblastomas	MCNU	Pilot Study	TNF-SAM2 80 × 10^4^ U/m^2^; MCNU 100 mg/m^2^ (IV); radiation (1.5Gy/day; 54-60Gy total)	8 week cycle. MCNU day 1. TNF-SAM2 day 3. TNF-SAM2 given weekly for 5 doses	4	12%	N/A	NR
**Fukushima 2003 [79]**	26	Malignant astrocytomas and glioblastomas	MCNU	Pilot Study	TNF-SAM2 80 × 10^4^ U/m^2^; MCNU 100 mg/m^2^ (IV); radiation (1.5Gy/day; 54-60Gy total)	8-12 week cycle. MCNU day 1. TNF-SAM2 day 3. TNF-SAM2 given weekly for 5 doses	4	17%	N/A	NR
^a^ All Studies used intravenous infusion for delivery of TNF-α. ^b^ Ojective response rate calculated using number of patients evaluable for response where available.^c^ Grade 3 or greater toxicities. TNF-α - tumor necrosis factor alpha. ORR - objective response rate. NR - not reported in study. N/A - not applicable for study design. MCNU - ranimustine.											

## FUTURE DIRECTIONS OF SYSTEMIC TNF-α

Translation of systemic TNF-α from research to clinic has been hampered by significant systemic toxicity and a lack of efficacy at MTD [43, 46]. Future directions for the development of TNF-α therapy rely on amelioration of the toxicity seen with systemic therapy and thereby increasing direct tumor response through higher TNF-α doses. Alternatively, the exploitation of novel mechanisms of action may increase efficacy and safety through indirect tumor effects.

Polyethylene glycol (PEG) conjugated proteins have shown increased retention and decreased immunogenicity *in vivo* [80]. Attempts to conjugate rhTNF-α with PEG has yielded a therapeutic with decreased toxicity and increased efficacy in murine preclinical models [81-84]. Thamm and colleagues conducted a phase I clinical trial of PEG-rhTNF-α in dogs with spontaneously occurring tumors [85]. Comparatively, Client-owned dogs provide an excellent model in which to develop novel anticancer agents. These dogs are genetically diverse, immunocompetent, share our environment and have similar types and size of tumors to people [86]. Interestingly, in this study the MTD of PEG-rhTNF-α was found to be 26.7μg/kg (approximately 815μg/m^2^) and 4 of 15 dogs treated had a partial tumor response. DLT was similar to that observed with unconjugated TNF-α, with hypotension and coagulopathy being observed. This study suggests that PEG-rhTNF-α may limit some of the undesirable toxicity seen with unconjugated TNF-α and allow for greater antitumor responses.

Asparagine-glycine-arginine conjugated to the N-terminus of TNF-α (NGR-TNF-α) specifically binds the aminopeptidase N (CD13) of tumor vasculature [87]. CD13 is required for the pathological development of vasculature in the disease and presents an ideal target to modulate the effect of chemotherapeutics [88, 89]. Preclinical studies of NGR-TNF-α showed synergism with doxorubicin, cisplatin, placitaxel and gemcitabine, increasing tumor penetration of cytotoxic compounds, anticancer efficacy and decreasing treatment associated toxicity [90]. Interestingly, increase in efficacy was seen *in vivo* but not *in vitro* with tumor cell lines, indicating that this synergism is due to an indirect effect of NGR-TNF-α on host vasculature [90]. A recent Phase Ib study of low-dose NGR-TNF-α with doxorubicin in advanced solid tumors demonstrated that this combination is well tolerated with no DLT observed [67]. A phase II dose of 0.8μg/m^2^ of NGR-TNF-α and 75mg/m^2^ of doxorubicin was recommended. The study provided hope for future development of TNF-α and doxorubicin combination therapy with 1 of 15 patients achieving a partial response and 10 of 15 patients with stable disease for a median duration of nearly 6 months.

An alternative concept for the use of TNF-α in the treatment of human cancers exists. Preclinical *in vivo* studies demonstrated that the uptake of radiolabeled liposomes in tumors was increased by approximately 6 fold in mice that were concomitantly treated with TNF-α [4]. The mechanism behind this enrichment is thought to be mediated through effects on the tumor vasculature and an enhanced-enhanced permeability and retention (E^2^PR) effect. *In vivo* experiments using the combination of TNF-α and liposomal doxorubicin showed a significantly increased survival benefit in tumor-bearing mice treated with the combination in comparison to mice treated with either TNF-α or liposomal doxorubicin alone. Although single-agent liposomal doxorubicin alone delayed tumor growth and led to improved survival, the tumors eventually grew back, whereas the combination treatment with TNF-α and liposomal doxorubicin led to a long-term survival in 80% of the treated animals. These findings are in accordance with previously published data showing improved treatment outcomes in rat osteosarcoma and murine melanoma tumor models that were treated with low-dose TNF-α plus liposomal doxorubicin in comparison to TNF-α plus free doxorubicin [5, 91]. The development of low-dose TNF-α and liposomal doxorubicin may provide unique synergy to increase efficacy and decrease toxicity of combination therapy. Clinical studies are necessary to establish the safety and efficacy of this approach. These studies are worthwhile considering the novel mechanism of synergism between TNF-α and liposomal doxorubicin.

## CONCLUSION

TNF-α has been proven an effective anticancer agent in *in vitro* and *in vivo* preclinical studies. Sadly, the promise of systemic TNF-α has, as of yet, not translated to a patient therapy and enthusiasm has been curbed due to the toxicity profile and lack of efficacy at MTD. Combination with chemotherapy in the setting of hyperthermic isolated limb perfusion has proven quite successful, based not only on a direct anti-proliferative effect of TNF-α, but also due to its ability to increase drug penetration into tumor tissue. The future development of systemic TNF-α as an anticancer treatment will rely on exploring ways to reduced systemic toxicity and exploit novel mechanisms of action to deliver greater efficacy simultaneously with decreased toxicity. A number of avenues are currently being explored based on promising preclinical and early clinical data. The novel concept of using systemic TNF-α to facilitate increased tumor penetration of liposomal chemotherapy seems particularly promising and worth exploring clinically.
